# New Biomarkers Probing Depth of Cell Senescence Assessed by Laser Scanning Cytometry

**DOI:** 10.1002/cyto.a.20983

**Published:** 2010-10-11

**Authors:** Hong Zhao, H Dorota Halicka, Frank Traganos, Ellen Jorgensen, Zbigniew Darzynkiewicz

**Affiliations:** Department of Pathology, Brander Cancer Research Institute, New York Medical CollegeValhalla, New York 10595

**Keywords:** image analysis, trichostatin A, maximal pixel of fluorescence intensity, nuclear area, EU incorporation, growth imbalance, cell morphometry, chromatin condensation, cell cycle, apoptosis, p21^WAF1^

## Abstract

The imaging analytical capabilities of laser scanning cytometer (LSC) have been used to assess morphological features considered to be typical of the senescent phenotype. The characteristic “flattening” of senescent cells was reflected by the decline in the density of staining (intensity of maximal pixel) of DNA-associated fluorescence [4,6-diamidino-2-phenylindole (DAPI)] paralleled by an increase in nuclear size (area). The decrease in ratio of maximal pixel to nuclear area was even more sensitive senescence biomarker than the change in maximal pixel or nuclear area, each alone. The saturation cell density at plateau phase of growth recorded by LSC was found to be dramatically decreased in cultures of senescent cells, thereby also serving as an additional marker. The induction of cyclin dependent kinase inhibitors p21^WAF1^ and p27^KIP1^ and γH2AX and activation of ATM markers of DNA damage response were measured in parallel with DNA/DAPI maximal pixel and nuclear area. These biomarker indices were expressed in quantitative terms by reporting them as a fraction of the respective controls. The effect of treatment of A549 and WI-38 cells with different concentrations of mitoxantrone (Mxt) and trichostatin A for various time periods was studied to assess the degree (depth) of cell senescence. Also assessed was the effect of 2-deoxy-d-glucose, the agent attenuating metabolic cell activity, on the depth of senescence induced by Mxt. A relationship between the ability of cells to synthesize RNA (incorporate 5-ethynyluridine) that leads to growth imbalance and induction of cell senescence was also studied. The data show that morphometric analysis of cellular attributes by LSC offers an attractive tool to detect cell senescence and measure its degree particularly in assessing effects of the factors that enhance or attenuate this process. This methodology is of importance in light of the evidence that cellular senescence is not only a biological process that is fundamental for organismal aging but also impedes formation of induced-pluripotent stem cells providing the barrier for neoplastic transformation and is the major mechanism of induction of reproductive cell death during treatment of solid tumors. © 2010 International Society for Advancement of Cytometry.

The goal of treatment of malignancies is containment of tumor growth either by prompt killing of the targeted tumor cells or by elimination of their reproductive potential. Apoptosis is frequently the mode of cell death on treatment with most antitumor modalities. Because induction of apoptosis involves complex interactions between many signaling pathways and arrangement of the highly sophisticated execution machinery of cell death, diverse antitumor strategies have been proposed to affect these interactions to preferentially kill tumor cells while sparing normal cells (1–5). Although cells in certain malignancies, particularly leukemias, are prone to undergo apoptosis, in many solid tumors, the mechanism of treatment-induced suppression of growth relies on irreversible impairment of cell reproductive capacity (“reproductive death”), which is often defined as “senescence-like growth arrest,” “accelerated senescence,” “premature senescence,” or “drug- or radiation-induced senescence” (5–10). Extensive mitogenic signaling and overexpression of certain oncogenes may also lead to proliferation arrest characterized by senescence-like features. The loss of reproductive potential of cells undergoing malignant transformation, whether through induction of apoptosis or senescence, plays an important role as one of the barriers to tumor development (7,11–16).

Identification of senescent cells relies on several markers. The most characteristic are morphological alterations (17,18). Senescent cells are described as having low saturation density at the plateau phase of growth, “flattened” appearance, enlarged, often irregular nuclei, and cytoplasmic granularities. An increase in cellular size is paralleled by an increase in nuclear and nucleolar size. There are numerous vacuoles in the cytoplasm, increased number of cytoplasmic microfilaments, the presence of large lysosomal bodies, and prominent Golgi apparatus (17–19). The increased lysosome mass is likely due to the presence of secondary lysosomes containing indigestible materials such as lipofuscins (18,20–22). Gross abnormalities are characteristics of nuclear chromatin of senescent cells. The most prominent is the presence of senescence-associated heterochromatic foci (SAHF), which are abundant in histone H3 modified at lysine 9 (K9M H3) and its binding partner heterochromatin protein 1 (HP1) (23–25). However, it should be pointed out that the presence of SAHF while observed in senescent WI-38 and IMR90 cells was not apparent in some other cell types (26).

A variety of molecular markers characterize cells undergoing senescence. The suppressed proliferation of senescent cells is, to a large degree, induced by overexpression of inhibitors of cyclin dependent kinases (CDKs) p21^WAF1^, p16^INK4A^, and/or p27^KIP1^ that may or may not be mediated by the tumor suppressor p53 (14,27–33). There is also persistent expression of markers of DNA damage response, particularly γH2AX (34–36). However, it should be stressed that among all biomarkers the most specific to senescent cells are: (i) the characteristic changes in cell morphology and (ii) the induction of senescence-associated β-galactosidase activity; the latter is considered to be the hallmark of cell senescence (37,38).

In this study, we have examined whether the senescence-associated alterations in cell morphology can be quantitatively assessed by laser scanning cytometer (LSC). LSC is the microscope-based analytical instrument that offers the combined advantages of flow cytometry and image analysis (39–41). It can analyze ∼100 cells per second, record a large number of the measured parameters including cell morphometric data, and preserve images of the analyzed cells (41). Our present data reveal that the very simple analysis of the nuclear size (area) combined with the intensity of maximal pixel of DNA associated fluorescence on its staining with 4,6-diamidino-2-phenylindole (DAPI) provide a convenient marker of the degree of cell senescence. The use of this marker made it possible to study the effects of several factors modulating the propensity of different cell types to undergo senescence, such as suppression of metabolic activity by 2-deoxy-d-glucose (2dG), capacity to continue cell growth (synthesize RNA), and importance of cell cycle phase. Using multiparameter analysis, we were able to correlate the induction of senescence with the expression of p21^WAF1^ and parameters of DNA damage response such as phosphorylation of H2AX and activation of ataxia telangiectasia mutated kinase (ATM).

## MATERIALS AND METHODS

### Cells, Cell Treatment

Human pulmonary adenocarcinoma A549 cells were purchased from American Type Culture Collection (ATCC #CCL-185, Manassas, VA). The cells were cultured in Ham's F12K medium with 2 mM l-glutamine adjusted to contain 1.5 g/l sodium bicarbonate (GIBCO/Invitrogen, Carlsbad, CA) and supplemented with 10% fetal bovine serum (FBS; GIBCO/Invitrogen). WI-38 fibroblasts were also from ATCC and were grown in MEM Eagle–Earle medium containing essential and nonessential amino acids, 2mM l-glutamine, and 10% FBS. Dual-chambered slides (Nunc Lab-Tek II Fisher Scientific, Pittsburgh, PA) were seeded with 1 ml of 10^5^ cells/ml cell suspension per chamber 48 h before exposure. All incubations were performed at 37°C in a humidified atmosphere of 5% CO_2_ in air. Trichostatin A (TSA) and 2dG were from Sigma–Aldrich (St. Louis, MO). Mitoxantrone (Mxt) was obtained from Lederle Laboratories (Pearl River, NY). Drug concentrations and other details are given in figure legends. At the end of the incubation, medium from each chamber was carefully aspirated and 1 ml of 1% fresh methanol-free formaldehyde in 1× phosphate buffered saline (PBS) was added to each chamber and the cells fixed by gently rocking the slides at room temperature for 15 min. Following aspiration of the fixative, the chamber slides were disassembled and the slides submerged in 70% ethanol. The fixed slides were stored at 4°C before analysis. The incorporation of the RNA precursor 5-ethynyluridine (EU) was detected by the “click” methodology (42), using the protocol and the reagents provided by Invitrogen/Molecular Probes (Carlsbad, CA).

### Immunocytochemical Detection of p21^WAF1^, p16^KIP1^, Phosphorylated Histone H2AX (γH2AX), and Activated ATM

After fixation, the cells were washed twice in PBS and treated on slides with 0.1% Triton X-100 (Sigma) in PBS for 15 min, and then in a 1% (w/v) solution of bovine serum albumin (BSA; Sigma) in PBS for 30 min to suppress nonspecific antibody (Ab) binding. The cells were then incubated in a 100 μl volume of 1% BSA containing 1:100 dilution of p21^WAF1^ or p16^KIP1^ Ab (Cell Signaling, Danvers, MA) or 1:200 dilution of phosphospecific (*Ser*139) γH2AX mAb (Biolegend, San Diego, CA) or 1:100 dilution of phosphospecific (*Ser*1981) ATM mAb (Cell Signaling) for 1.5 h at room temperature or overnight at 4°C. The secondary fluorochrome-tagged Abs used were either Alexa Fluor 488 tagged Ab (Invitrogen/Molecular Probes, at 1:100 dilution) or Alexa Fluor 633 Ab (Invitrogen/Molecular Probes, at 1:100 dilution). Before fluorescence measurement on the LSC, the cells were counterstained with 2.8 μg/ml DAPI (Sigma) in PBS for 15 min. Other details of cell incubations with the primary and secondary Ab were presented before (43–45).

### Measurement of Cell Fluorescence by LSC

Cellular green or far red immunofluorescence (IF) representing binding of the respective phosphospecific Abs and the blue emission of DAPI stained DNA was measured using an LSC (iCys® Research Imaging Cytometer; CompuCyte, Westwood, MA) and standard filter settings; fluorescence was excited with 488 nm argon, helium–neon (633 nm), and violet (405 nm) lasers (39–41). The intensities of maximal pixel and integrated fluorescence were measured and recorded for each cell. At least 3,000 cells were measured per sample. The standard deviation was estimated based on Poisson distribution of cell populations. Other details are given in figure legends.

## RESULTS

**Figure 1:**
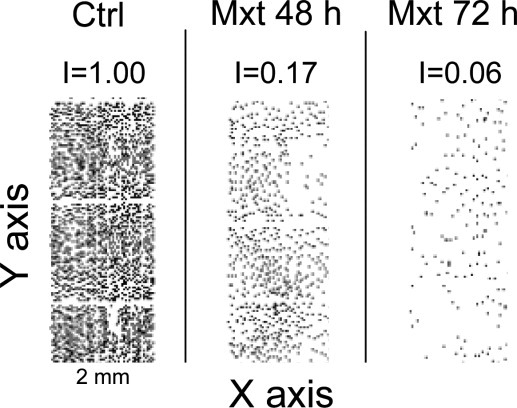
Decreased cell saturation density at confluence in cultures of A549 cells growing in the presence of Mxt. The cells we untreated (Ctrl) or grown in the presence of 2 nM Mxt for 48 or 72 h in chamber slide-cultures. The cells were fixed, stained with DAPI, and their location on the slides was recorded by LSC (36,37). The observed decrease in cell density in the Mxt-treated cultures can be presented as a fraction (index, I) of cell density (I = 1.00) in the untreated culture of the same age.

**Figure 2:**
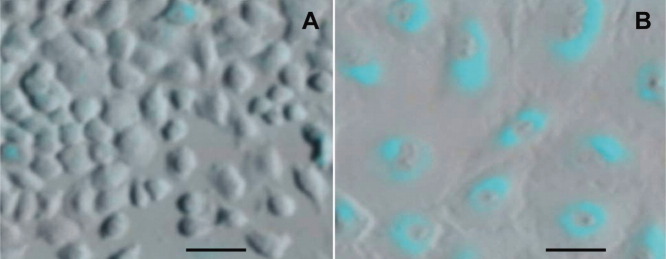
Induction of senescence-associated β-galactosidase activity in A549 cells growing for 72 h in the presence of 2 nM Mxt. Images of cells growing in the absence (A) and presence of Mxt (B) recorded by LSC (Research Imaging Cytometer iCys). Fifty Micrometer bars mark the length scale.

Different approaches have been used to induce senescence of A549 or WI-38 cells. In one set of experiments, senescence of human lung carcinoma A549 cells was induced by their exposure to low concentration (2 nM) of the DNA topoisomerase II inhibitor Mxt, an anthracenedione that binds to DNA by intercalation (46). Following growth for 48 h in the presence of Mxt, cell morphology was altered as the cells acquired features characteristic of senescence. The LSC analysis of the cell density on slides on which cells were growing revealed that the saturation density at confluence was distinctly lower compared with the control cells growing in parallel cultures, and the density was further decreasing with time of treatment with Mxt (depth of senescence; Fig. 1). Numerically, when the cell density in the treated cultures was expressed as a fraction of that in the untreated ones of the same age (1.00), it was apparent that after exposure to Mxt for 48 and 72 h there were only 0.16 and 0.06 cells at confluence, respectively. Figure 2 illustrates the induction of the senescence-associated β-galactosidase activity (37,38) in A549 cells growing in the presence of Mxt for 72 h. It is quite evident that the β-galactosidase positive cells are larger and have much lower saturation density on the slide.

**Figure 3:**
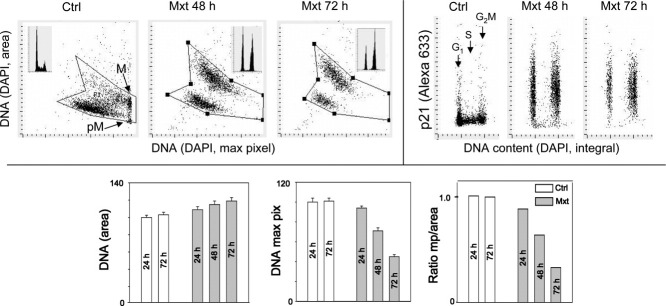
LSC-assisted morphometric analysis of nuclear changes and expression of p21^WAF1^ of A549 cells in cultures treated with Mxt. The cells were untreated (Ctrl) or treated with 2 nM Mxt for 24, 48, or 72 h, their DNA was stained with DAPI and p21^WAF1^ was detected immunocytochemically (Alexa 633—indirect Ab). Intensity of maximal (max) pixel of DNA/DAPI reports degree of chromatin condensation and in untreated cells has the highest value and marks mitotic (M) and immediately postmitotic (pM) cells. In the senescing cells, while nuclear area increases, the intensity of maximal pixel decreases likely due to the “flattening” of the cell. The insets in the top left panels show DNA frequency histograms of cells from the respective cultures. The bar plots at the bottom panels show mean values (±standard deviation) of nuclear DNA/DAPI area, DNA/DAPI maximal pixel and the ratio of maximal pixel to nuclear area. The ratio of maximal pixel/nuclear area of the Mxt-treated cells is expressed as a fraction of such ratio of the respective control (1.0).

Taking into an account the change in morphology of cells undergoing senescence revealed by the increased nuclear size and the remarkable cell “flattening”, we expected that the morphometric analysis of the nucleus by LSC would provide a sensitive marker of such a change. Indeed, when the nuclear size represented by the area of the DNA-associated DAPI fluorescence was plotted versus the maximal pixel of DNA/DAPI fluorescence on bivariate distribution scatterplots (Fig. 3), it was apparent that senescent cells in the Mxt-treated cultures had increased area and distinctly decreased intensity of maximal pixel of DNA/DAPI fluorescence. As DNA/DAPI area was increased and the intensity of maximal pixel decreased in senescent cells, the ratio of maximal pixel to area (mp/area) was decreased to an even greater degree during treatment with Mxt and progressively with time. Concurrent analysis of the integral of intensity of DAPI fluorescence and immunofluorescence of p21^WAF1^ revealed the arrest in G_1_ and G_2_M phases of the cell cycle, which was paralleled by a remarkable increase in expression of p21^WAF1^ (Fig. 3). When the initially treated with 2 nM Mxt for 72 h A549 cells were cultured in Mxt-free medium for 10 subsequent days, there was no evidence of formation of cell colonies (not shown). This observation provided verification of the loss of their reproductive capability.

**Figure 4:**
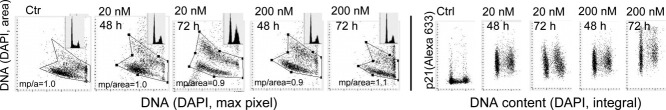
Effect of Mxt at 20 nM and 200 nM concentration on morphometric features of A549 cells and on p21^WAF1^ expression. The cells were grown in the presence of Mxt for 48 or 72 h; their DNA was stained with DAPI, and p21^WAF1^ was detected immunocytochemically. The ratio of maximal pixel of DAPI fluorescence intensity/nuclear area (mp/area) in the Mxt-treated cells is expressed as a fraction of such ratio of the untreated cells (Ctrl; 1.0).

As the ratio of intensity of maximal pixel of DAPI fluorescence to DAPI area (mp/area) was shown to be a sensitive biomarker of cell senescence, we tested the effects of higher concentrations of Mxt, shown before to lead to apoptosis rather than senescence on prolonged treatment in cultures (47). As it is evident in Figure 4, exposure of A549 cells to 10-fold and 100-fold higher Mxt concentration caused an increase in expression of p21^WAF1^ and some arrest in the cell cycle, but essentially had no effect on mp/area ratio, which varied between 0.9 and 1.1.

**Figure 5:**
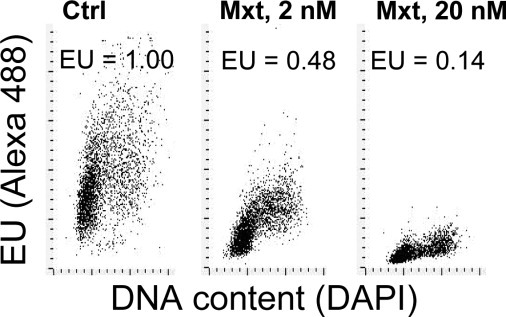
Effect of Mxt at 2 and 20 nM concentration on ability of A549 cells to synthesize RNA. The cells were untreated (Ctrl) or treated with 2 and 20 nM Mxt for 4 h, then the RNA precursor EU was added for 30 min. The incorporation of EU was detected using a copper (l)-catalyzed cycloaddition reaction (“click” chemistry) with fluorescent azide (44). The mean fluorescence intensity for all cells in the Mxt treated cultures is expressed as a fraction of the mean fluorescence of the untreated cells.

The induction of cell senescence appears to require cell cycle arrest and ongoing or even accelerated metabolic activity (cell growth), particularly ribosome biogenesis that is known to be mediated by the mammalian target of rapamycin (mTOR) pathway (48). Therefore, we have tested the ability of cells to synthesize RNA at Mtx concentration that induced senescence (2 nM) and compared it with Mtx concentration that did not lead to senescence (20 and 200 nM). As it is evident from the data shown in Figure 5, incorporation of the RNA precursor EU, detected by the “click” chemistry methodology (42), was suppressed by 52 and 86% in cells treated with 2 and 20 nM Mxt, respectively. The suppression was even greater at 200 nM concentration of Mxt (not shown).

**Figure 6:**
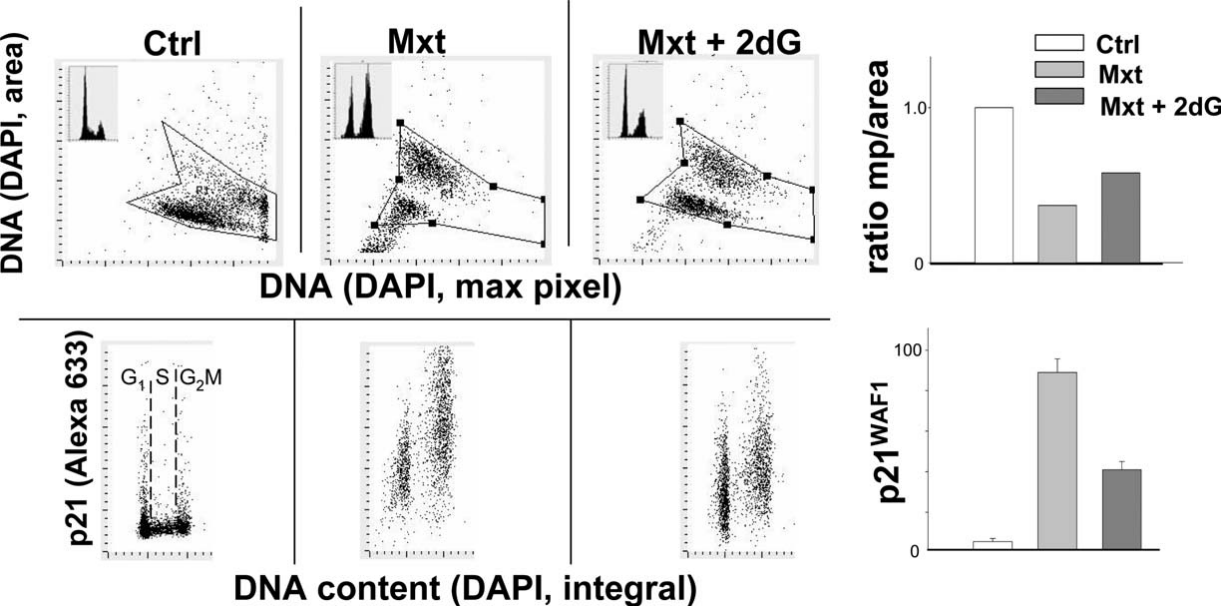
Effect of 2dG on depth of senescence induced by Mxt. A549 cells were grown in the absence (Ctrl) or presence of 2 nM Mxt alone (Mxt) or 2 nM Mxt and 5 mM 2dG (Mxt + 2dG) for 72 h. Their DNA was stained with DAPI, and p21^WAF1^ was detected immunocytochemically. Note attenuation of the ratio mp/area and p21^WAF1^ in cultures growing in the presence of 2dG compared to Mxt alone.

Although activation of cell growth and metabolic rate enhances the rate of induction to senescence and “depth” of senescence, the suppression of growth and of metabolic rate has an opposite effect (8,28–31,49,50). The glucose antimetabolite 2dG decreases oxidative phosphorylation and was shown to lower the constitutive DNA damage response, the reporter of DNA damage induced by endogenously generated reactive species generated during oxidative metabolism (51,52). We have tested whether the suppression of the cell's metabolic rate by 2dG can affect the depth of senescence induced by Mxt reflected by nuclear morphometry as measured by LSC. As is show in Figure 6, treatment of A549 cells with Mxt in the presence of 2dG diminished both the degree of reduction of intensity of maximal pixel fluorescence of DAPI (DNA) and the decrease of mp/area ratio compared with cell growth in the presence of Mxt alone. The expression of p21^WAF1^ paralleled the changes in nuclear morphometry showing that the degree of its induction in cells growing in the presence of Mxt and 2dG was distinctly suppressed compared with cells growing in the presence of Mxt alone (Fig. 6).

**Figure 7:**
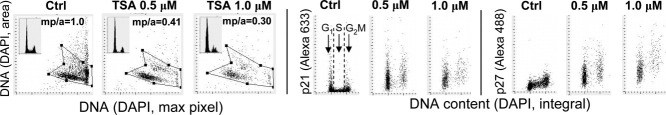
Induction of senescence of A549 cells by TSA. The cells were grown in absence (Ctrl) or presence of TSA at 0.5 or 1.0 μM concentration for 48 h. Their DNA was stained with DAPI, expression of p21^WAF1^ and p27^KIP1^ was detected immunocytochemically and all parameters were measured by LSC.

One of the frequently used means of induction of cell senescence involves their growth in the presence of histone deacetylase (HDAC) inhibitors (53,54). Thus, we studied the effect of exposure of A549 cells to one such inhibitor, TSA, by measuring the changes in intensity of maximal pixel DNA/DAPI fluorescence and nuclear area in the cells growing in its presence (Fig. 7). Consistent with the data on the induction of senescence of A549 cells by the DNA-damaging drug Mxt (Fig. 3), dramatic reduction in the intensity of the maximal pixel fluorescence concurrent with the induction of p21^WAF1^ and p27^KIP1^ have been seen in cells treated with 0.5 and 1.0 μM of TSA. The change in the ratio of mp/area was more pronounced at 1.0 μM (mp/area = 0.30) than at 0.5 μM (mp/area = 0.41) concentration of TSA.

**Figure 8:**
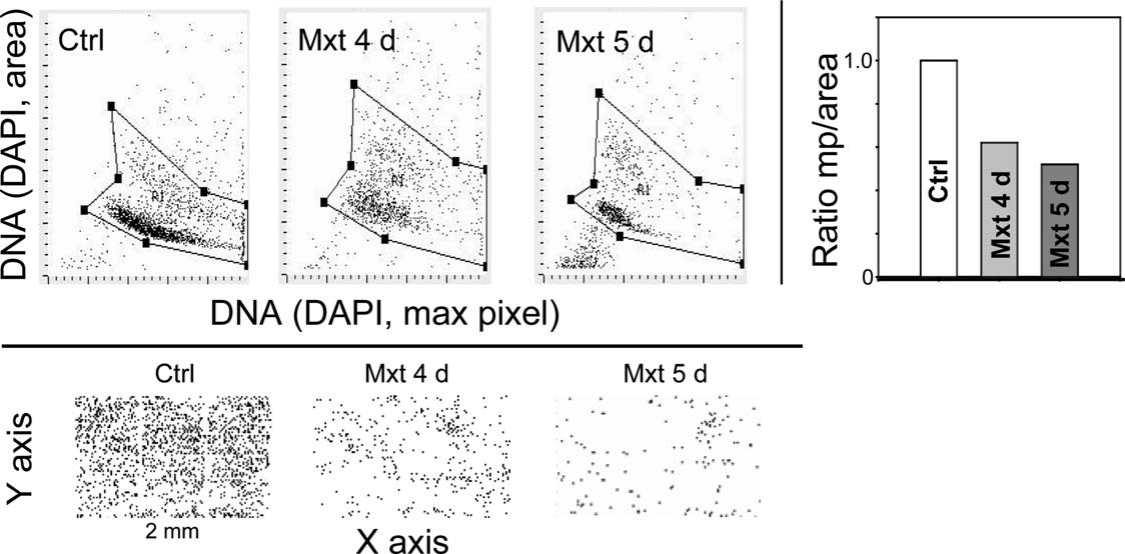
Induction of senescence of WI-38 cells represented by the decrease in intensity of maximal pixel DAPI fluorescence and mp/area ratio following treatment with 2 nM Mxt for 4 and 5 days. Bottom panels show saturation density of cells at confluence in the respective cultures, with I = 1.0, I = 0.31 and I = 0.19 in Ctrl, after 4 and 5 days of treatment with Mxt, respectively.

As mentioned previously, one of the characteristic features of chromatin senescent changes is the presence of SAHF (23–25). Heterochromatin is a condensed state of chromatin with DNA more compressed per unit of space compared with euchromatin. Therefore, one would expect that SAHF may have increased intensity of maximal pixel of DNA/DAPI fluorescence and the approach we used to identify senescent cells based on analysis of DAPI/DNA maximal pixel by LSC may not be applicable to cells with SAHF. The presence of SAHF was described predominantly in senescing fibroblasts WI-38 (23–25). We have tested whether induction of senescence in WI-38 cells can be detected by the biomarker based on the intensity of DAPI/DNA maximal pixel. WI-38 cells when treated with 2 nM Mxt and viewed under the fluorescence microscope following staining with DAPI were indeed characterized by the presence of SAHF (not shown). However, as it is evident from the data shown in Figure 8 the senescing WI-38 cells, similar to A549, show a distinct decrease in intensity of maximal pixel of DNA/DAPI fluorescence and marked reduction of the mp/area ratio. Thus, the presence on SAHF does not interfere with the use of the maximal pixel intensity of DAPI fluorescence as a marker of cell senescence.

**Figure 9:**
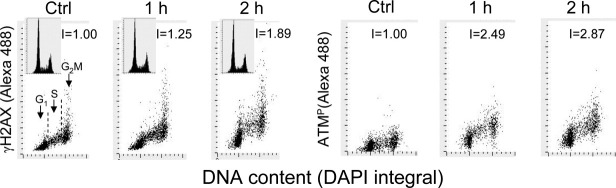
Early events of DNA damage response marked by induction of γH2AX and phosphorylation of ATM on Ser1981 (ATM^P^) after treatment of A549 cells with 2nM Mxt for 1 or 2 h. The index I represents the level of induction of γH2AX and ATM-S1981^P^ markers in Mxt-treated cells related to their constitutive level in the untreated (Ctrl) cells (I = 1.00).

Induction of cell senescence is one of the facets of DNA damage response, and it provides the barrier preventing tumor development (9,11–16) as well as impeding formation of induced-pluripotent stem cells (iPSCs) and potential initiators of neoplastic transformation (55). Among the early events of DNA damage response are activation of ATM by its phosphorylation on Ser1981 and phosphorylation of histone H2AX on Ser139, changes that can be detected by and measured by LSC (56,57). We have tested whether the low (2 nM) concentration of Mxt that induces cell senescence after 48–96 h of exposure triggers these early events of DNA damage response. The data shown in Figure 9 demonstrates that exposure of A549 cells to 2 nM Mxt for only 1 or 2 h caused a distinct increase in the level of phosphorylation of these proteins. In cells treated with Mxt for 2 h, the increase in expression of γH2AX and ATM-S1981P was by 89 and 187%, respectively, above the level of expression of these phosphoproteins seen in the untreated cells (Ctrl). The latter represents constitutive phosphorylation of these proteins that in large part reports DNA damage response to the damage triggered by endogenous metabolically generated oxidants (52,57).

## DISCUSSION

Induction of senescence of A549 and WI-38 cells has been studied to explore the analytical capability of LSC for identification of senescent cells phenotype and quantitative assessment of the senescence process. The data obtained by this approach provided some interesting information. To explain why senescence was induced at low (1 or 2 nM) but not at higher (20 or 200 nM) concentration of Mxt (Fig. 4), we have measured the ability of cells to synthesize RNA at these concentrations. The “click” chemistry approach (42) was used to measure incorporation of EU by individual cells to be measured by LSC. The data show that while there was a rather modest attenuation of RNA synthesis at 2 nM (∼50%), much more dramatic suppression was seen at 20 nM (>86%; Fig. 5) and 200 nM (>95%) Mxt concentrations. These observations are consistent with the notion that ongoing RNA synthesis (cell growth) is needed to drive the cells toward senescence (49,57–59). When the cell cycle progression is halted, whether by a DNA damaging drug (Mxt; Fig. 3) or HDAC (TSA; Fig. 8), cell growth has to continue for the cells to develop the senescent phenotype (50). In fact, prolonged maintenance of unbalanced growth caused by suppression of DNA replication by variety of inhibitors was shown to lead to development of the senescent phenotype as well as to upregulation of senescence-associated genes (58). However, in all the instances of cell cycle arrest, when inhibition of cell growth rate was attenuated either by: (i) reduction of protein synthesis or suppression of extracellular signal-regulated kinase/mitogen-activated protein (ERK/MAP) kinase pathway (58), (ii) blocking mTOR pathway (49), (iii) inhibition of glycolysis by 2dG (Fig. 6), or (iv) caloric restriction (50), the propensity of cells to undergo senescence was distinctly reduced. In contrast, oncogene-driven acceleration of growth rate promotes induction of cell senescence (8,59). The present data are in support of most of the earlier observations linking cell growth as an essential event for the induction of senescence of cells that are arrested in the cell cycle.

The availability of the biomarkers probing cell death is very much needed both in basic research as well as in clinical studies. Although there is vast array of different markers of apoptotic cells (reviews, refs.^60^–^62^), fewer are available to detect senescent cells. Particularly, there is a lack of methods that can quantitatively assess the degree (“depth”) of cell senescence. However, it becomes more and more apparent that while apoptosis is common during treatment of leukemias, senescence is the more predominant mode of death of tumor cells during treatment of solid tumors. In this study, using the morphometric analytical capabilities of LSC, we describe a simple approach to measure the alterations in nuclear and chromatin structure that accompany cell senescence. The observed decrease in intensity of maximal pixel of DNA/DAPI fluorescence and the increase in nuclear area measured by LSC is a consequence of a change of cell geometry namely its extensive flattening of the senescent cell. Because the thickness of the nucleoplasm is diminished compared with the nonsenescent cell, the intensity of DNA-associated fluorescence (DAPI) per unit of nuclear image (pixel) also is diminished. Thus, the biomarker based on analysis of nuclear area and intensity of maximal pixel of DNA-associated fluorescence is a sensitive reporter of the characteristic change in cell phenotype considered to be the hallmark of senescence.

Another attribute of the senescence phenotype, also considered to be its hallmark, is the low saturation density in cultures at the plateau phase of cell growth. Analysis of cell density on slides/cultures is provided automatically by LSC and is routinely plotted on the *X*-axis versus *Y*-axis scatterplots (Figs. 1 and 8). Thus, cell densities in cultures suspected to senesce can be simply compared with the densities of the control culture of the same age to obtain a quantitative parameter characterizing degree (depth) of quiescence based on analysis of saturation density at confluency.

Analysis of intensity of maximal pixel of DNA-associated (DAPI) fluorescence and nuclear area, especially when expressed as a ratio of mp/area, made it possible not only to identify senescent cells but also provides the means to express and compare the degree (depth) of senescence in quantitative terms. This has been done by normalizing (standardizing) the change in nuclear morphometry in senescing cells through expressing this as a fraction of such ratio of the respective nonsenescent control cells. We have used this approach to compare and quantify the effect of treating cells for different lengths of time and to different concentrations of inducers or inhibitors of cell senescence.

Taking advantage of multiparameter and multispectral analysis provided by LSC (iCys Research Imaging Cytometer), which has three-color laser illumination and four channels of fluorescence measurement, the morphometric nuclear changes (maximal pixel and area) were measured in parallel with analysis of the induction of p21^WAF1^ and p27^KIP1^. As a result, it was possible to measure the correlation between the morphometric nuclear change and the expression of these CDK inhibitors on a cell by cell basis as well as to measure other attributes of the cell and to correlate them with the senescence biomarker. It should be noted that measurement of fluorescence intensity by LSC is accomplished with photomultipliers that offer a wide dynamic range of fluorescence intensity measurement, exceeding that of image analyzers that use charge-coupled device cameras (39–41).

## CONCLUSIONS

Organismal aging is a consequence of individual cells undergoing senescence. The induction of cell senescence appears also to be the critical mode of cell death in solid tumors during chemotherapy or radiotherapy. Cell senescence is also a key element of the tumor suppressor pathways including the pathway modulating formation of the iPSCs (55,63). Therefore, there is a need for quantitative biomarkers that can be used to assess cell senescence *in vitro* and *in vivo*. This data demonstrate that morphometric analysis of cellular attributes by LSC offers an attractive tool to detect cell senescence and measure its degree particularly in assessing the factors accelerating or delaying this process. By relying on analysis of change in geometry of cell nucleus and spatial distribution of DNA in chromatin, this approach may provide a sensitive biomarker that has a potential to be used on biopsy- and tissue section-specimens.
